# Genomic Characterization of Extended-Spectrum Cephalosporin-Resistant *Salmonella enterica* in the Colombian Poultry Chain

**DOI:** 10.3389/fmicb.2018.02431

**Published:** 2018-10-26

**Authors:** Luis Ricardo Castellanos, Linda van der Graaf-van Bloois, Pilar Donado-Godoy, Maribel León, Viviana Clavijo, Alejandra Arévalo, Johan F. Bernal, Dik J. Mevius, Jaap A. Wagenaar, Aldert Zomer, Joost Hordijk

**Affiliations:** ^1^Department of Infectious Diseases and Immunology, Faculty of Veterinary Medicine, Utrecht University, Utrecht, Netherlands; ^2^Colombian Integrated Program for Antimicrobial Resistance Surveillance – Coipars, Corporación Colombiana de Investigación Agropecuaria - Corpoica, Mosquera, Colombia; ^3^Dirección Técnica de Inocuidad e Insumos Veterinarios, Instituto Colombiano Agropecuario - ICA, Bogotá, Colombia; ^4^Department of Biological Sciences, Los Andes University, Bogotá, Colombia; ^5^Department of Bacteriology and Epidemiology, Wageningen Bioveterinary Research, Lelystad, Netherlands

**Keywords:** Latin America, chicken, *S*. Paratyphi B d-tartrate positive, *S*. Heidelberg, *S*. Java, MLST, pMLST

## Abstract

*Salmonella enterica* serovars have been isolated from Colombian broilers and broiler meat. The aim of this study was to investigate the diversity of ESBL/pAmpC genes in extended-spectrum cephalosporin resistant *Salmonella enterica* and the phylogeny of ESBL/pAmpC-carrying *Salmonella* using Whole Genome Sequencing (WGS). A total of 260 cefotaxime resistant *Salmonella* isolates, obtained between 2008 and 2013 from broiler farms, slaughterhouses and retail, were included. Isolates were screened by PCR for ESBL/pAmpC genes. Gene and plasmid subtyping and strain Multi Locus Sequence Typing was performed *in silico* for a selection of fully sequenced isolates. Core-genome-based analyses were performed per ST encountered. *bla*_CMY−2−like_ was carried in 168 isolates, 52 carried *bla*_CTX−M−2_ group, 7 *bla*_SHV_, 5 a combination of *bla*_CMY−2−like_-*bla*_SHV_ and 3 a combination of *bla*_CMY−2−like_-*bla*_CTX−M−2_ group. In 25 isolates no ESBL/pAmpC genes that were screened for were found. WGS characterization of 36 selected strains showed plasmid-encoded *bla*_CMY−2_ in 21, *bla*_CTX−M−165_ in 11 and *bla*_SHV−12_ in 7 strains. These genes were mostly carried on IncI1/ST12, IncQ1, and IncI1/ST231 plasmids, respectively. Finally, 17 strains belonged to *S*. Heidelberg ST15, 16 to *S*. Paratyphi B variant Java ST28, 1 to *S*. Enteritidis ST11, 1 to *S*. Kentucky ST152 and 1 to *S*. Albany ST292. Phylogenetic comparisons with publicly available genomes showed separate clustering of Colombian *S*. Heidelberg and *S*. Paratyphi B var. Java. In conclusion, resistance to extended-spectrum cephalosporins in *Salmonella* from Colombian poultry is mainly encoded by *bla*_CMY−2_ and *bla*_CTX−M−165_ genes. These genes are mostly associated with IncI1/ST12 and IncQ1 plasmids, respectively. Evolutionary divergence is observed between Colombian *S*. Heidelberg and *S*. Paratyphi B var. Java and those from other countries.

## Introduction

*Salmonella* Enteritidis and *S*. Typhimurium have been reported as the most frequent serovars causing salmonellosis in humans worldwide (Hendriksen et al., 2011). According to data collected in the European Union (EU) in 2015 and the United States (USA) in 2013, *S*. Enteritidis accounted for 46 and 15% of *Salmonella* infections and *S*. Typhimurium for 16 and 13%, respectively (Centers for Disease Control and Prevention (CDC), 2016; European Food Safety Authority (EFSA) and European Centre for Disease Prevention and Control (ECDC), 2016). Likewise, *S*. Typhimurium and *S*. Enteritidis are the most isolated serovars from human cases in Colombia. Their overall prevalence among human isolates between 2005 and 2011 was 32 and 28%, respectively (Rodríguez et al., 2017).

Among foods of animal origin, poultry products (e.g., eggs) have been primarily associated with *S*. Enteritidis infections in humans while *S*. Typhimurium infections are associated with a wider range of sources including pork, beef and poultry products (Mughini-Gras et al., 2014; Antunes et al., 2016). Nevertheless, in broilers and chicken meat the prevalence of serotypes other than *S*. Enteritidis and *S*. Typhimurium have been on the rise over the last 2 decades (van Pelt et al., 2003; Foley et al., 2011; Wagenaar et al., 2013). Baseline studies of *Salmonella* performed between 2005 and 2006 in broiler chickens in the EU showed *S*. Infantis, *S*. Mbandaka, and *S*. Hadar to be highly prevalent (European Food Safety Authority (EFSA), 2007). In the year 2006, *S*. Infantis, *S*. Enteritidis and *S*. Paratyphi B d-tartrate positive (here referred as *S*. Paratyphi B variant Java) were the most frequent serovars in broiler meat in the EU (European Food Safety Authority (EFSA), 2007). More recently, *S*. Infantis, *S*. Enteritidis, *S*. Mbandaka, and *S*. Ohio were the most prevalent serovars in broilers and broiler meat in 2015 (European Food Safety Authority (EFSA) and European Centre for Disease Prevention and Control (ECDC), 2016). In North America, *S*. Kentucky, *S*. Enteritidis, *S*. Typhimurium, *S*. Heidelberg, and *S*. Infantis are reported as the most prevalent serovars in broilers and ground chicken meat in the USA (United States Department of Agriculture (USDA) - Food Safety and Inspection Service (FSIS), 2013) while in Canada, *S*. Heidelberg, *S*. Kentucky and *S*. Enteritidis are the most prevalent in broilers and broiler meat (Public Health Agency of Canada, 2016). In Colombia, baseline studies performed by the Colombian integrated program for antimicrobial resistance surveillance (Coipars), demonstrated *S*. Paratyphi B var. Java, and *S*. Heidelberg to be the most prevalent serovars in broiler farms and meat at retail. Serovar distribution at farm level was 76 and 23%, respectively (Donado-Godoy et al., 2012b), and at the retail level 45 and 19%, respectively (Donado-Godoy et al., 2014). A similar distribution of *S*. Paratyphi B var. Java and *S*. Heidelberg, has been reported in chicken meat at retail in Guatemala (35 and 16% respectively) (Jarquin et al., 2015) and broilers at slaughter in Venezuela (62 and 31% respectively) (Boscán-Duque et al., 2007).

Isolates of *S*. Paratyphi B var. Java and *S*. Heidelberg are often multi drug resistant (Denny et al., 2007; Dutil et al., 2010; Antunes et al., 2016; Liakopoulos et al., 2016) and have been associated with carriage of plasmid-mediated extended spectrum β-lactamases (ESBLs) and plasmid associated AmpC β-Lactamases in poultry (pAmpC) (Antunes et al., 2016). For instance, *S*. Paratyphi B var. Java isolates from poultry in Europe have been found carrying the ESBL gene *bla*_CTX−M−1_ and the pAmpC gene *bla*_CMY−2_ (Doublet et al., 2014; Mevius et al., 2015; Veldman et al., 2016). In turn, *S*. Heidelberg has been associated to *bla*_CMY−2_ in North America (Andrysiak et al., 2008; Folster et al., 2012) and poultry products imported from South America into Europe (Liakopoulos et al., 2016). In South America *S*. Heidelberg has been associated with *bla*_CTX−M−2_ (Antunes et al., 2016). To date, no data of the genetic determinants of Extended Spectrum Cephalosporin (ESC) resistance in *Salmonella* from broilers and chicken meat in Colombia are available. These data are highly relevant to understand the epidemic spread of ESBL/pAmpC-producing *Salmonella* in poultry resulting in frequent occurrence in chicken meat in multiple countries.

Serotyping has traditionally been used for the epidemiological investigation of *Salmonella*, but does not provide information about the evolutionary relatedness of strains. Sequence based methodologies such as Multi Locus Sequence Typing (MLST) and Whole Genome Sequencing (WGS) have been proposed as a replacement of serotyping to identify evolutionary and epidemiological relatedness (Achtman et al., 2012; Ashton et al., 2016; Nadon et al., 2017). Additionally, information on the genetic basis of antimicrobial resistance (AMR) and plasmids harboring AMR genes can be readily obtained from WGS assemblies (Zankari et al., 2012; Carattoli et al., 2014). Altogether, the objectives of this study were to investigate the diversity of ESBL/pAmpC genes and encoding plasmids found in ESC-resistant *S. enterica* from broilers and broiler meat in Colombia and to determine the genetic relatedness with *Salmonella* strains from other countries using MLST and core-genome alignments.

## Materials and methods

### Isolates of *salmonella enterica*

The isolates included in this study originated from different cross-sectional baseline studies conducted between 2008 and 2013 at different stages during the development of Coipars. In these studies, non-clinical samples were obtained from three different levels of poultry production in Colombia: Broiler farms (Donado-Godoy et al., 2012b), broilers at slaughter (Donado-Godoy et al., 2015b) and broiler meat at retail (Donado-Godoy et al., 2012a, 2014, 2015a). The methodology for sampling, random isolation (i.e., without antimicrobials during enrichment) of *Salmonella* and antimicrobial susceptibility testing with the BD Phoenix automated system, was previously described in detail for the studies in broiler farms, broilers at slaughter and broiler meat at retail mentioned above. Previous results of the prevalence of *Salmonella* and resistance to ESC at the different levels of poultry production, are summarized in Table 1.

**Table 1:** Prevalence and origin of *Salmonella* isolates used in this study, distribution of ESBL/pAmpC genes and selection of isolates for WGS.

	**Number of samples**	***Salmonella*-positive (%)**	**ESBL/pAmpC-producing *Salmonella* (%) ^a^**	***bla*_CMY-2-like_**	***bla*_CTX-M-2_ group**	***bla*_SHV_ family**	***bla*_CMY-2-like_-*bla*_CTX-M-2_ group**	***bla*_CMY-2-like_-*bla*_SHV_ family**
Farms	74	28 (38)	17 (61)	16/2^b^	–	–	–	1/1^b^
2008	65	23	17	16	–	–	–	1
2009	9	5	–	–	–	–	–	–
Slaughter	644	140 (22)	82 (59)	54/4^b^	25/2^b^	–	2/2^b^	1/1^b^
2008	40	5	3	3	–	–	–	–
2012	251	76	39	31	8	–	–	–
2013	353	59	40	20	17	–	2	1
Retail	1554	410 (26)	136 (33)	98/7^b^	27/6^b^	7/7^b^	1/1^b^	3/3^b^
2009	179	38	11	11	–	–	–	–
2010	387	156	35	29	1	4	–	1
2011	823	170	76	49	22	2	1	2
2012	165	46	14	9	4	1	–	–
Total	2272	578 (25)	235 (41)	168/13 ^b^	52/8^b^	7/7^b^	3/3^b^	5/5^b^

a Based on PCR screening of ESBL/pAmpC genes.

b *ESBL/pAmpC-positive samples/Selected for whole genome sequencing*.

For the present study, all available *S. enterica* isolates (*n* = 673) were considered. The 673 isolates belonged to 578 production flocks, 28 flocks from broiler farms, 140 from slaughterhouses and 410 from retail (Table 1). Next, ESC-resistant isolates of *Salmonella* were selected based on suspected phenotypic resistance to cefotaxime, as previously measured with the BD Phoenix *(*Donado-Godoy et al., 2012b*)* and interpreted using the CLSI 2014 clinical breakpoints for Enterobacteriaceae (MIC ≥ 4 μg/ml) (Clinical and Laboratory Standards Institute (CLSI), 2014). For all flocks with multiple ESC-resistant isolates, each first isolate was selected to make sure we included epidemiologically unrelated isolates only.

As a result, a total of 260 isolates were selected for ESC-resistance characterization. The selected isolates from farms originated from drag swabs of fecal material from the floor of broiler houses and fresh feces directly from the chicken. These isolates originated from 19 broiler farms (*n* = 19) (Donado-Godoy et al., 2012b). Isolates at slaughter, originated from cecal content and carcass rinse from 32 slaughterhouses (*n* = 84) (Donado-Godoy et al., 2015b). At retail, isolates originated from carcass rinse from 143 retail suppliers (Donado-Godoy et al., 2012a, 2014) and chicken thighs meat from 8 retail suppliers (*n* = 157) (Donado-Godoy et al., 2015a). The isolates originated from 18 out of 32 departments (i.e. provinces) of Colombia. Together, these 18 departments are responsible for more than 90% of the chicken population in the country. A map of Colombia with the location of these departments is available in Supplementary Figure 1.

Information related to the origin of the samples and the prevalence of *Salmonella* is shown in Table 1.

### PCR screening of ESBL/pAmpC genes

For the present investigation, the 260 isolates selected as mentioned above, were screened by PCR for the presence of ESBL/pAmpC genes families using previously described primers for *bla*_TEM_ and *bla*_SHV_ families, *bla*_CMY−2−like_ and *bla*_CTX−M_ family (Dierikx et al., 2010); *bla*_CTX−M−1_ group (Carattoli et al., 2008); *bla*_CTX−M−2_ group (Jiang et al., 2006); *bla*_CTX−M−8_ group (Hopkins et al., 2006); *bla*_CTX−M−9_ group (Paauw et al., 2006); *bla*_OXA−1−like_, *bla*_OXA−2−like_ and *bla*_OXA−10−like_ (Voets et al., 2011).

### Selection of strains and WGS

Based on the diversity of gene families found after PCR screening, a representative selection of isolates was made and further subjected to WGS. Due to its high prevalence, a random selection was made using the Random function in Microsoft® Excel to assign random numbers to isolates positive for *bla*_CMY−2−like_ and *bla*_CTX−M−2_ group. The size of selection was fixed to the square root of the number of resulting positive strains for these genes. This resulted in 13 *bla*_CMY−2−like_- and 8 *bla*_CTX−M−2_ group-carrying isolates. In addition and due to their low prevalence, all positive isolates for *bla*_SHV_ family and combinations of *bla*_CMY−2−like_-*bla*_SHV_ family and *bla*_CMY−2−like_-*bla*_CTX−M−2_ group were included. An overview of the selection of isolates is shown in Table 1.

Isolation of genomic DNA from selected isolates was performed using the UltraClean® Microbial DNA Isolation Kit (Mo Bio-Qiagen, USA). WGS was performed on Illumina MiSeq and NextSeq platforms (Illumina, USA) using 2 × 250-bp reads and 2 × 150-bp reads, respectively. Genomes were assembled with SPAdes v3.10.1 (Bankevich et al., 2012).

### *In silico* characterization of ESBL/pAmpC gene variants

Subtyping of ESBL/pAmpC gene variants was performed using ResFinder 2.1 (Zankari et al., 2012). Investigation of resistance genes with an identity percentage < 100% was done using BLAST 2.6.0+ (Camacho et al., 2009).

### *In silico* characterization of ESBL/pAmpC-carrying plasmids

Plasmid content of selected strains was investigated using PlasmidFinder 1.3 and pMLST 1.4 (Carattoli et al., 2014).

Identification of plasmids associated to the ESBL/pAmpC genes was based on co-localization on the same contig as resulted from ResFinder and PlasmidFinder analysis. Since this was not possible for all genomes, transformation of plasmids harboring each of the ESBL/pAmpC variants identified with ResFinder was performed together with selective culturing as described before (Castellanos et al., 2017). This was done to obtain the plasmid types identified with PlasmidFinder in transformed *Escherichia coli* DH10B harboring the different ESBL/pAmpC variants identified. Afterwards, the sequences of the transformed plasmids were used as reference to map against the genomes of the selected strains. To this purpose, transformants were sequenced with Illumina MiSeq and NextSeq sequencing as described above. Chromosomal contigs in transformants were detected and removed by mapping against the *E. coli* DH10B genome sequence (GenBank accession number: CP000948.1) using BLAST. The remaining contigs were considered to be part of the ESBL/pAmpC carrying plasmid and were used as a reference. Next, the contigs of the initially sequenced selected strains were aligned to the obtained reference plasmid sequences using MUSCLE (Edgar, 2004). Alignments were made on selected strains according to the ESBL/pAmpC gene variants they harbored. Resulting aligned contigs were selected and considered as newly inferred ESBL/pAmpC-carrying plasmids.

### *In silico* MLST and serotype prediction

To determine the population structure of the selected ESC-resistant *Salmonella*, 7-gene MLST at the strain level was performed *in silico* with MLST 1.8 (Larsen et al., 2012). Serotype was predicted using the *Salmonella In Silico* Typing Resource (SISTR) (Yoshida et al., 2016). Whole genome phylogenetic analyses were performed for *S*. Heidelberg ST15 and *S*. Paratyphi B var. Java ST28. Given the limited number of isolates (*n* = 1), this was not performed for *S*. Enteritidis ST11, *S*. Kentucky ST152 or *S*. Albany ST292.

### Collection of publicly available genomes for phylogenetic comparisons

Genome sequences of *S*. Paratyphi B var. Java ST28, were downloaded from Enterobase^1^ (last accessed: 12-Sept-2017) for comparison. Likewise, sequences of *S*. Heidelberg ST15 were obtained from Enterobase^1^ (last accessed: 11-Jan-2017). Only genomes with data available for their year of isolation, country of origin and source were considered for *S*. Heidelberg ST15. For both *S*. Paratyphi B var. Java ST28 and *S*. Heidelberg ST15, the genomes were collected disregarding their susceptibility to 3^rd^ generation cephalosporin's or to other antimicrobials. Additionally, the quality of genomes obtained in this study and the downloaded genomes was assessed with CheckM (Parks et al., 2015). Only genomes with >98% completeness score, when compared against the set of genomic markers for *S. enterica*, were included. MLST designation was amended using a custom BLAST-based tool^2^.

### Core genome phylogenetic analysis

Whole genome analysis was performed by a core-genome alignment using Parsnp v1.2 (Treangen et al., 2014). Recombination regions in the core genome alignment were detected and filtered using Gubbins (Croucher et al., 2015). Phylogenetic maximum likelihood (mid-point rooted) trees were constructed with the recombination-filtered core genomes alignments using FastTree2^3^ (Price et al., 2010) and visualized with FigTree^4^.

### Data availability

The obtained genome sequences of the *Salmonella* strains selected for WGS (Table 2) and those of the transformed *E. coli* DH10B strain with the ESBL/pAmpC-carrying plasmids used as reference have been deposited in the short-read archive of the ENA under Project Number: PRJEB23610.

**Table 2:** Origin of the *Salmonella* isolates selected for WGS and results of the characterization of their ESBL/pAmpC genes, plasmids, and strains.

**Source**	**Year**	**Strain**	**Location** **(Department)**	**Accession number** **ENA**	**β-lactam resistance genes^a^**	**ESBL/pAmpC-harboring plasmid^c^**	***S. enterica*** **Serovar**	***S. enterica*** **MLST**
Farm	2008	SSXXXV.4.C1	Santander	ERS2017899	*bla_CMY−2_*	IncI1/ST12	*S*. Java	ST28
		SSIII.4.C2	Santander	ERS2017900	*bla_CMY−2_*-${\mathrm{bla}}_{\text{SHV}-129}^{b}$-*bla*_TEM−1A_	IncI1/ST12	*S*. Java	ST28
		SXXI.1.C5	Cundinamarca	ERS2017901	*bla_CMY−2_*	IncI1/ST12	*S*. Java	ST28
Slaughter	2012	FBOG8	Bogotá	ERS2017931	*bla_CMY−2_*	IncI1/ST12	*S*. Java	ST28
		FSAN161	Santander	ERS2017935	*bla_CTX−M−165_*-*bla*_TEM−1B_	IncQ1	*S*. Heidelberg	ST15
		FSAN236	Santander	ERS2017936	*bla_CTX−M−165_*-*bla*_TEM−1B_	IncQ1	*S*. Heidelberg	ST15
		FBOG7	Bogotá	ERS2017938	*bla_CMY−2_*	IncI1*^d^*	*S*. Java	ST28
	2013	FSUC414	Sucre	ERS2017932	*bla_CMY−2_-bla_CTX−M−165_*-*bla*_TEM−1B_	IncI1/ST12-IncQ1*^e^*	*S*. Heidelberg	ST15
		FCAR509	Bolívar	ERS2017933	*bla_CMY−2_*	IncI1/ST12	*S*. Heidelberg	ST15
		FANT596	Antioquia	ERS2017934	*bla_CMY−2_*-*bla*_SHV−129_-*bla*_TEM−1B_	IncI1/ST12	*S*. Java	ST28
		FVAL369	Valle del Cauca	ERS2017937	*bla_CMY−2_*	IncI1/ST12	*S*. Heidelberg	ST15
		FPAS506	Nariño	ERS2017939	*bla_CMY−2_-*bla*_CTX−M−165_*	IncI1/ST12-Non - typeable*^e^*	*S*. Heidelberg	ST15
Retail	2010	UGBOG4	Bogotá	ERS2017904	*bla_CMY−2_-bla_SHV−12_*	IncI1/ST12-Non - typeable*^e^*	*S*. Java	ST28
		UGBOG316	Bogotá	ERS2017908	*bla_SHV−12_*	Non-typeable	*S*. Heidelberg	ST15
		UGBOG327	Bogotá	ERS2017909	*bla_SHV−12_*	IncI1/ST231	*S*. Java	ST28
		UGBOG339	Bogotá	ERS2017910	*bla_SHV−12_*	IncI1/ST231	*S*. Java	ST28
		UGBOG340	Bogotá	ERS2017911	*bla_SHV−12_*	IncI1/ST231	*S*. Java	ST28
	2011	UGBAR394	Atlántico	ERS2017912	*bla_CMY−2_-*bla*_CTX−M−165_*-*bla*_TEM−1B_	IncI1/ST12-IncQ1*^e^*	*S*. Heidelberg	ST15
		UGBAR434	Atlántico	ERS2017913	*bla_CMY−2_*	IncI1/ST12	*S*. Albany	ST292
		UGCAR507	Bolívar	ERS2017914	*bla_CTX−M−165_*-*bla*_TEM−1B_	IncQ1	*S*. Heidelberg	ST15
		UGVAL515	Valle del Cauca	ERS2017915	*bla_CMY−2_*-*bla*_SHV−129_	IncI1*^d^*	*S*. Heidelberg	ST15
		UGBUC832	Santander	ERS2017916	*bla*_SHV−129_	-	*S*. Heidelberg	ST15
		UGCUC851	Norte de Santander	ERS2017917	*bla_CMY−2_*	IncI1/ST12	*S*. Java	ST28
		UGCUC867	Norte de Santander	ERS2017918	*bla_CTX−M−165_*-*bla*_TEM−1B_	IncQ1	*S*. Heidelberg	ST15
		UGARA888	Arauca	ERS2017919	*bla_CMY−2_*	IncI1/ST12	*S*. Enteritidis	ST11
		UGIBA933	Tolima	ERS2017920	*bla_SHV−12_*	IncI1/ST231	*S*. Java	ST28
		UGPER971	Risaralda	ERS2017921	*bla_CMY−2_*	IncI1/ST12	*S*. Java	ST28
		UGBOG1024	Bogotá	ERS2017922	*bla_CMY−2_*	IncI1/ST12	*S*. Java	ST28
		UGPAS1097	Nariño	ERS2017923	*bla_CMY−2_*	IncI1/ST12	*S*. Kentucky	ST152
		UGBUC1112	Santander	ERS2017924	*bla_CTX−M−165_*	Non-typeable	*S*. Heidelberg	ST15
		UGBUC1123	Santander	ERS2017925	*bla_CTX−M−165_*-*bla*_TEM−1B_	IncQ1	*S*. Heidelberg	ST15
		UGBAR1160	Atlántico	ERS2017926	*bla_CTX−M−165_*	IncQ1	*S*. Heidelberg	ST15
		UGBAR1170	Atlántico	ERS2017927	*bla_CMY−2_*-*bla*_SHV−129_-*bla*_TEM−1B_	IncI1/ST12	*S*. Java	ST28
		UGBAR1187	Atlántico	ERS2017928	*bla_CTX−M−165_*-*bla*_TEM−1B_	IncQ1	*S*. Heidelberg	ST15
	2012	UGBOG1279	Bogotá	ERS2017929	*bla_SHV−12_*	Non-typeable	*S*. Heidelberg	ST15
		UGBOG1280	Bogotá	ERS2017930	*bla_CMY−2_*	Non-typeable	*S*. Java	ST28

a The genes present in the contigs selected for plasmid characterization have been underlined.

b blaSHV-129 was non-transferable after electroporation experiments.

c The plasmids carrying the β-lactam resistance genes have been underlined.

d These plasmids missed one allele from the pMLST scheme.

e *Characterization of the two ESBL/pAmpC genes was performed in separate plasmid contigs*.

## Results

### PCR screening of ESBL/pAmpC genes

After PCR screening of the 260 isolates, 235 isolates were positive for the genes screened for. 168 were positive for *bla*_CMY−2−like_, 52 for *bla*_CTX−M−2_ group, 7 for *bla*_SHV_ family, 5 harbored a combination of *bla*_CMY−2−like_-*bla*_SHV_ family and 3 a combination of *bla*_CMY−2−like_-*bla*_CTX−M−2_ group. In 48 isolates, *bla*_TEM_ was co-located with *bla*_CMY−2−like_, *bla*_CTX−M−2_ group or *bla*_SHV_. In 25 isolates no ESBL/pAmpC genes that were screened for were encountered.

The distribution of positive samples for *Salmonella* according to their source, year and ESBL/pAmpC genes is shown in Table 1.

### Selection of strains for WGS

After PCR characterization, a random selection of *bla*_CMY−2−like_-positive isolates (*n* = 13) and *bla*_CTX−M−2_ group (*n* = 8) together with all positive isolates for *bla*_SHV_ family (*n* = 7), *bla*_CMY−2−like_-*bla*_SHV_ family (*n* = 5) and *bla*_CMY−2−like_-*bla*_CTX−M−2_ group (*n* = 3) were included for WGS. In total, 36 isolates were selected and subjected to WGS. The list of selected isolates and the results of the characterization of ESBL/pAmpC genes, plasmid types, serotypes, and strain MLST based on WGS is shown in Table 2.

### Characterization of ESBL/pAmpC gene variants

After WGS characterization of resistance genes, 13 strains harbored *bla*_CMY−2_, 8 *bla*_CTX−M−165_, 6 *bla*_SHV−12_ and 1 *bla*_SHV−129_. Additionally, three harbored the combination of *bla*_CMY−2_-*bla*_CTX−M−165_, four *bla*_CMY−2_-*bla*_SHV−129_ and one *bla*_CMY−2_-*bla*_SHV−12_ (Table 2). All accompanying *bla*_TEM_ variants were identified as *bla*_TEM−1A_ or *bla*_TEM−1B_.

### *In silico* characterization of ESBL/pAmpC-carrying plasmids

Co-localization of ESBL/pAmpC and plasmid replicon genes in the same contig was observed for 17 out of 36 strains selected for WGS. For the remaining strains, co-localization was determined by analyzing selected contigs harboring ESBL/pAmpC and plasmid replicon genes using MUSCLE. Genes conferring resistance to other antimicrobials only co-localized with *bla*_CTX−M−165_-harboring contigs, not with other ESBL/pAmpC genes (Supplementary Table 1). In detail, one transformant was obtained carrying either a *bla*_CMY−2_-, *bla*_CTX−M−165_- or *bla*_SHV−12_-harboring plasmid. The plasmids isolated from strains UGBOG4 (*bla*_CMY−2_), UGBAR1160 (*bla*_CTX−M−165_) and UGBOG327 (*bla*_SHV−12_) (Supplementary Table 1) were used as a reference to map against the genomes of all selected strains. The *bla*_SHV−129_ gene present in five selected strains was not transferable by transformation from any of the strains, suggesting chromosomal localization of this gene.

After characterizing the plasmid contigs of the selected strains, 18 of 21 *bla*_CMY−2_-carrying plasmids were found to belong to IncI1/ST12, 1 was non-typeable based on the PCR Based Replicon Typing (PBRT) scheme used in PlasmidFinder (Carattoli et al., 2014). Two were designated IncI1, but the plasmid from strain UGVAL515 lacked the *pilL* allele and the plasmid from FBOG7 lacked the *sogS* allele. Nonetheless, these two plasmids remained single-allele variants of IncI1/ST12. For *bla*_CTX−M−165_, 9 of 11 plasmids harbored the IncQ1 plasmid-replicon and 2 remained non-typeable. Four of seven *bla*_SHV−12_ plasmids belonged to IncI1/ST231 and three remained non-typeable (Table 2 and Supplementary Table 1).

### Strain MLST, serotype characterization, and core genome phylogeny

**Figure 1:**
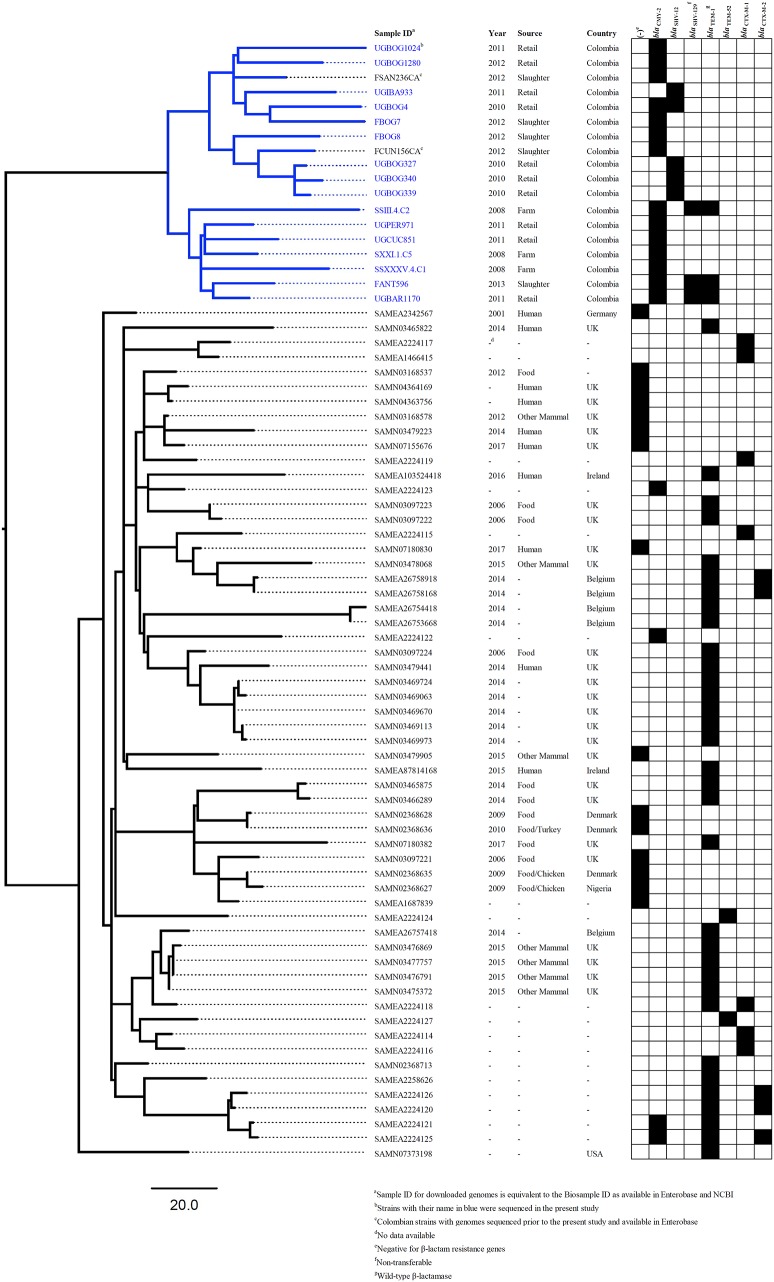
Core-genome phylogeny of *S*. Java ST28 using the genomes obtained in the present study and those available in Enterobase^1^ (last accessed: 12-Sept-2017).

**Figure 2:**
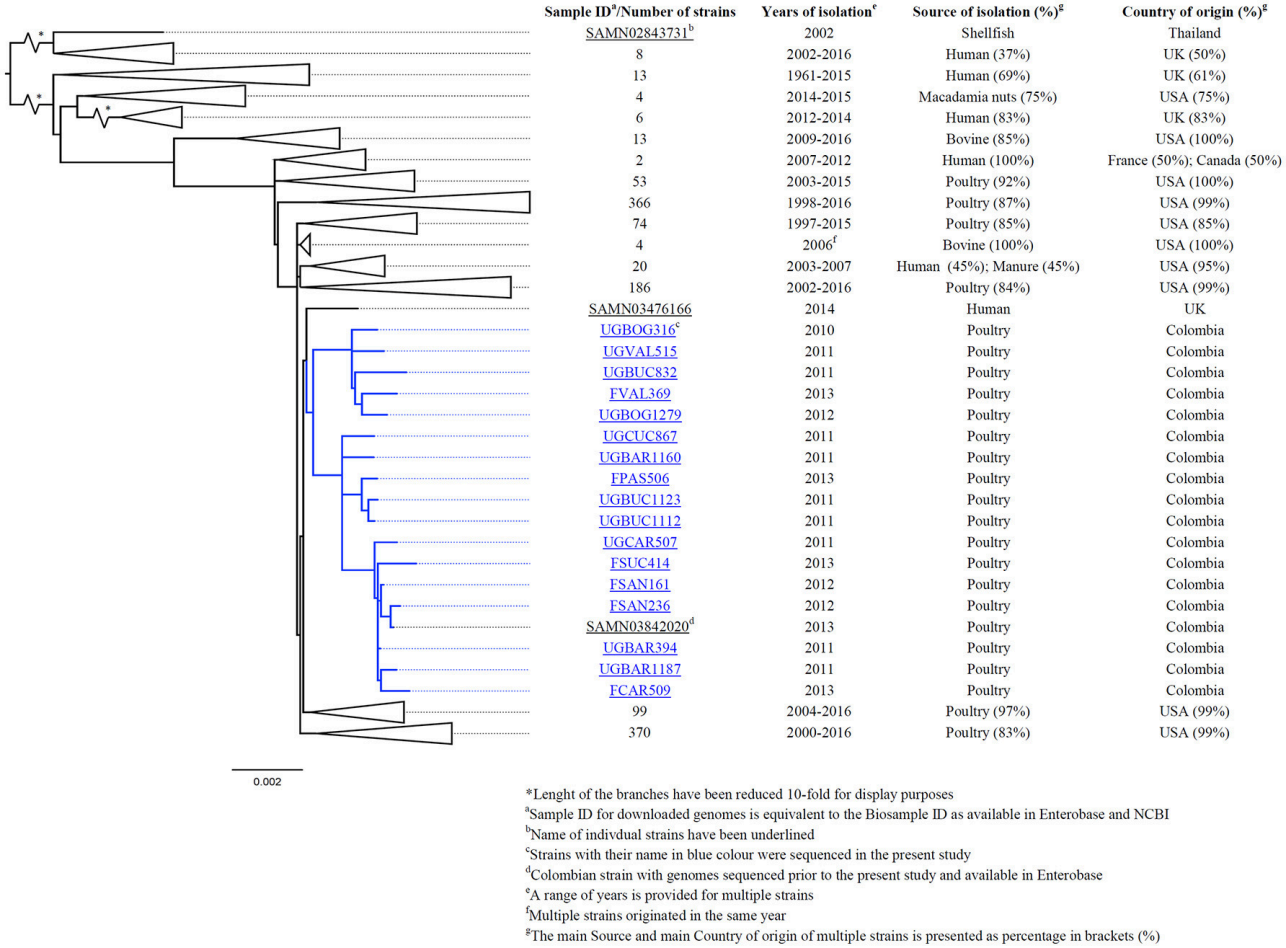
Core-genome phylogeny of *S*. Heidelberg ST15 using genomes obtained in this study and those with complete metadata selected from Enterobase^1^ (last accessed: 11-Jan-2017).

After using 7-gene MLST and the *Salmonella In Silico* Typing Resource (SISTR), 17 strains belonged to *S*. Heidelberg ST15, 16 to *S*. Paratyphi B var. Java ST28, 1 to *S*. Enteritidis ST11, 1 to *S*. Kentucky ST152 and 1 to *S*. Albany ST292 (Table 2). Further, whole genome analysis was performed for ST28 and ST15 isolates. For the phylogenetic analysis, additional genomes for ST28 (*n* = 60) and ST15 (*n* = 1221) were selected from Enterobase.warwick.ac.uk disregarding their characteristics of susceptibility to 3rd generation cephalosporins and used to construct the phylogenetic maximum likelihood trees. All Colombian genomes belonging to ST28 and ST15 formed a single cluster in the phylogenetic analysis. Phylogenetic trees for ST28 and ST15 with data regarding the source, year and country of the strains, are shown in Figures 1 and 2 respectively. No clustering related to the presence or absence of an ESBL/pAmpC gene is observed in Figure 1, suggesting the observed clustering is related to the geographical origin of *S*. Paratyphi B var. Java ST28 strains and not to the presence of an AMR gene. Likewise, in Figure 2, a cluster of *S*. Heidelberg ST15 strains originating from Colombian poultry is observed. Furthermore, the Colombian strains from ST28 and ST15 were disseminated in multiple departments within the country. A map of Colombia with the location of origin of ST28 and ST15 isolates selected for WGS is available in Supplementary Figure 2. An additional table with the metadata of strains selected for the construction of the ST28 and ST15 phylogenies is available as Supplementary Table 2.

## Discussion

In summary, *bla*_CMY−2_, *bla*_CTX−M−165_, *bla*_SHV−12_, and *bla*_SHV−129_ are described as the most prevalent ESBL/pAmpC genes conferring resistance to ESC in *S. enterica* isolated from the Colombian poultry chain between 2008 and 2013. According to the objectives of the present study, the collection of isolates served to reflect maximum diversity of ESBL/pAmpC genes in different years, departments and levels of the poultry production chain in Colombia.

The finding of *bla*_CMY−2_ as the main cause of ESC resistance is comparable to a previous report of ESBL/pAmpC-producing *E. coli* in Colombian poultry. In that study, it was encountered a prominent association of this gene with IncI1/ST12 plasmids (Castellanos et al., 2017). Those results suggested occurrence of horizontal gene transfer of this plasmid lineage between heterogeneous *E. coli* STs. The results from the present study, suggest that transfer of *bla*_CMY−2_-IncI1/ST12 plasmids between *E. coli* and *Salmonella* is also likely to occur, and could be considered a driver of the frequent occurrence of this resistance gene along the poultry chain. This particular gene-plasmid association has been described in *E. coli* from Brazil (da Silva et al., 2017), *Salmonella* from the USA (Folster et al., 2010) and *E. coli* and *Salmonella* from Europe (Accogli et al., 2013; Smith et al., 2015), suggesting an epidemiological link between the presence of *bla*_CMY−2_-IncI1/ST12 and poultry from different countries. Nevertheless, WGS of these plasmids is necessary to assess the level of genetic relatedness among them and estimate the potential transmission of these plasmids between *E. coli* and *Salmonella*.

After PCR screening, *bla*_CTX−M−2_ group was found to be the most prevalent ESBL gene among Colombian isolates. After subtyping a selection of these isolates with WGS, these genes were found to be *bla*_CTX−M−165_. To date, this variant has been solely reported in an isolate of *Klebsiella pneumoniae* from a urine sample in Chile and reported in 2016 (Accession number: KP727572) without further epidemiological records. Noticeably, all *bla*_CTX−M−165_-positive strains, alone or in combination with other *bla* genes, were identified in *S*. Heidelberg ST15. In our collection of isolates comprising the years 2008 to 2013, *bla*_CTX−M−165_ is only detected from the year 2010 onwards (Table 1). Not taking into account the potential bias that could occur by having isolates comprising a period of time no longer than 5 years, this finding may suggest a recent introduction of this gene in Colombian poultry, and until 2013, is limited to *S*. Heidelberg ST15. However, analysis of recently collected isolates of *S. enterica* and other Enterobacteriaceae is necessary to confirm this hypothesis.

After electroporation experiments, *bla*_SHV−129_ remained non-transferable and no plasmid markers were identified in its harboring contigs, which ranged in size between 2100 and 8594 bp. Therefore, it is likely that this gene is chromosomally located. Furthermore, the gene was found in two different serovars, *S*. Paratyphi B var. Java ST28, and *S*. Heidelberg ST15. It can be hypothesized that its transfer could be associated to an integrative or transposable element. Initial screening of transposases and Insertion Sequences (IS) using BLAST (Siguier, 2006) on the contigs harboring these genes detected several IS families flanking the sequences inside the contigs. Nevertheless, given the restricted size of the contigs no definite association with a unique IS element (e.g., AMR-associated IS) was possible. In such a case, complementing the short-read WGS data with additional data obtained through long-read sequencing is necessary to confirm the chromosomal location of this gene and its association to a particular mobile genetic element. This approach could also be used for further characterization of ESBL/AmpC-plasmids that were non-typeable according to the PBRT scheme used in PlasmidFinder, which could have also been affected by the limitations of genome and plasmid assembly of short-reads.

As mentioned previously, ESC and non-ESC *S*. Paratyphi B var. Java and *S*. Heidelberg were reported as the most prevalent serovars in the Colombian poultry chain (Donado-Godoy et al., 2012b, 2014). In the present study, investigation of resistance to cefotaxime showed a total of 235 (41%) resistant isolates from which, 17 (61%) originated from farms, 82 (59%) from slaughterhouses and 136 (33%) from retail (Table 1). Noteworthy, the prevalence of resistance diminishes from one level of production to the other. From previous studies, *S*. Paratyphi B var. Java and *S*. Heidelberg were the most prevalent serovars encountered at the farm level (Donado-Godoy et al., 2012b) and a larger diversity of serovars was found at retail, with more than 10 different serovars isolated repeatedly (Donado-Godoy et al., 2014). As observed in the present study, strains belonging to *S*. Paratyphi B var. Java and *S*. Heidelberg had a higher prevalence of ESC-resistance in comparison to the other serovars. These results indicate that the higher prevalence of *S*. Paratyphi B var. Java and *S*. Heidelberg, accounted in large part for the higher prevalence of ESC–resistance at the farm level and the presence of different serovars resulted in the reduction of resistance along the production chain, which is reflected in the lower prevalence of ESC-resistance at retail.

As anticipated, the analysis of *Salmonella* strains using MLST in addition to the resolution provided by WGS data has proven to be very useful in showing clustering of Colombian strains belonging to *S*. Paratyphi B var. Java ST28. According to the phylogenetic analysis including ESBL/pAmpC-positive and–negative strains (Figure 1), the clustering seems to occur independently of ESC-susceptibility and may be related to the geographical origin of the strains. Whether the cluster of Colombian *S*. Paratyphi B var. Java ST28 represents a particular separate lineage circulating in the country, or is present elsewhere in Latin America is a question that requires further investigation. At the time of publication of the present study no genomes of ST28 from other Latin American countries were publicly available and the comparisons within the region were limited to the strains we sequenced and analyzed.

In conclusion, resistance to ESC in *S. enterica* from Colombian poultry is mainly caused by *bla*_CMY−2_ and *bla*_CTX−M−165_ genes. These genes are mostly associated with IncI1/ST12 and IncQ1 plasmids, respectively. The resolution provided by WGS was appropriate to assess the evolutionary divergence of strains from Colombian poultry belonging to *S*. Paratyphi B var. Java ST28. Dissemination of ESBL/pAmpC genes in *Salmonella* is mainly due to the carriage of plasmids encoding these genes in strains belonging to *S*. Paratyphi B var. Java ST28 and *S*. Heidelberg ST15.

## Author contributions

JW, PD-G, JH, AZ, DM, LG-vB, and LC contributed to the design of the study. LG-vB and AZ performed the formal analysis. JW Contributed funding acquisition. LG-vB, PD-G, ML, VC, AA, JB, AZ, and LC conducted material and data collection. JW, PD-G, and AZ provided biological, laboratory and computational resources. JH, JW, DM, and AZ supervised the study. LC wrote the original draft. JH, JW, DM, LG-vB, and AZ critically reviewed the manuscript.

### Conflict of interest statement

The authors declare that the research was conducted in the absence of any commercial or financial relationships that could be construed as a potential conflict of interest.

## Footnotes

^1^https://enterobase.warwick.ac.uk/

^2^https://github.com/tseemann/mlst

^3^http://meta.microbesonline.org/fasttree/

^4^http://tree.bio.ed.ac.uk/software/figtree/
