# Supplementary figures and images for: Genomic Characterization of Extended-Spectrum Cephalosporin-Resistant Salmonella enterica in the Colombian Poultry Chain
Source: Front Microbiol. 2018 Oct 26;9:2431. doi: 10.3389/fmicb.2018.02431 (PMC6232905; doi:10.3389/fmicb.2018.02431)

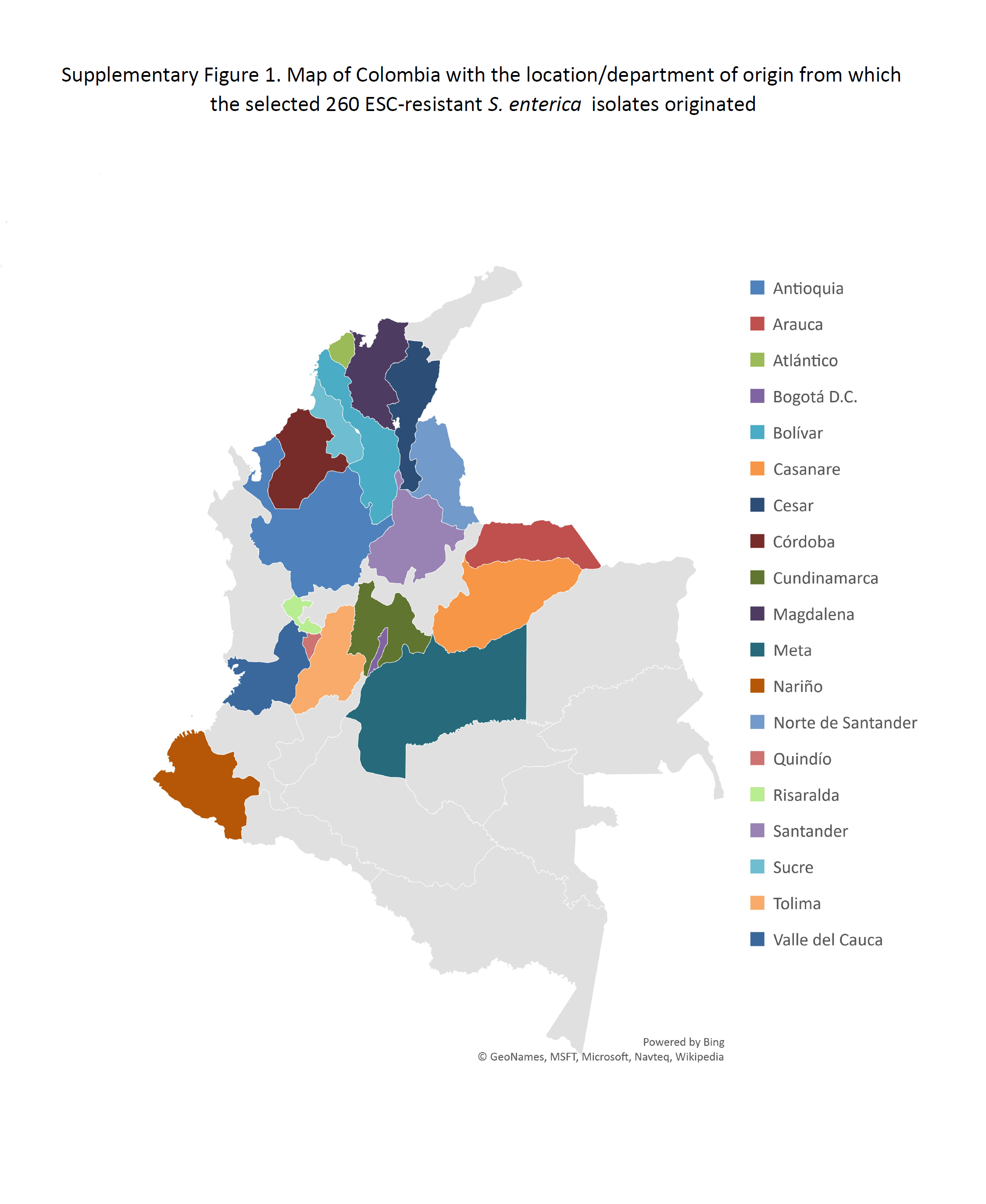

Supplement: Supplementary file 3 [file Image_1.TIF]

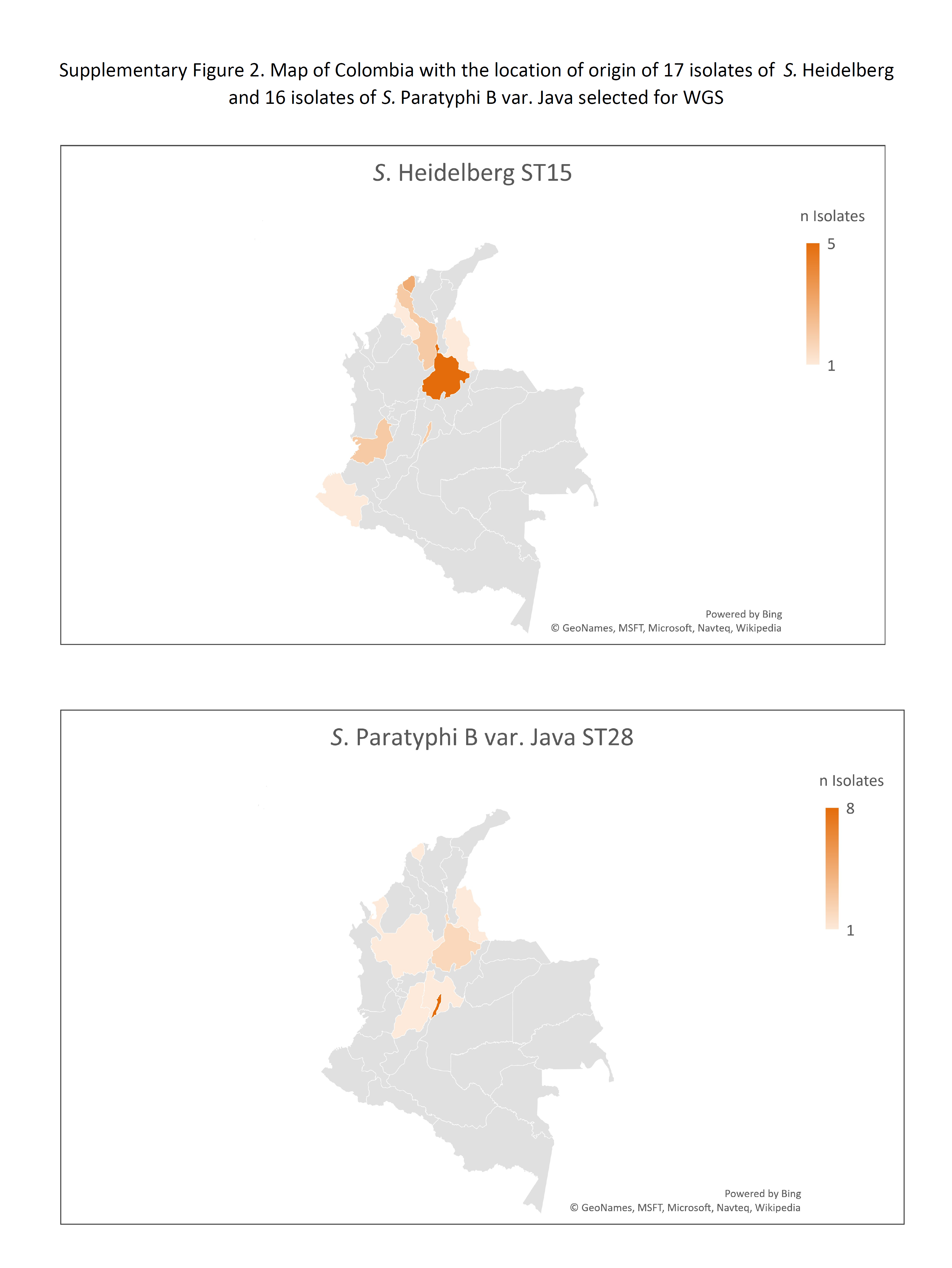

Supplement: Supplementary file 4 [file Image_2.TIF]
